# Supplementary material for: Chromosome-level assembly of the gray fox (Urocyon cinereoargenteus) confirms the basal loss of PRDM9 in Canidae
Source: G3 (Bethesda). 2024 Feb 15;14(4):jkae034. doi: 10.1093/g3journal/jkae034 (PMC10989890; doi:10.1093/g3journal/jkae034)
Supplement: jkae034_Supplementary_Data [file jkae034_supplementary_data.docx]

# **Supplementary Information**

Human_NP_064612.2 2 SPEKSQEESPEEDTERTERKPMV

+ + +

TAVPTDPLFAHPKALGYLHPGQG

grayfox_Scaffold_6__1_c 2 aggcagcctgccagtgtcccgcgGTGCGTG Intron 1

cctccacttcacactgatacgag<0-----[71 : 7328]

atccacttttctatgacactaga

Human_NP_064612.2 25 -KDAFKDISIYFTKEEWAEMGDWEKTRYRNVKRNYNALITI

KDAFK+ISIYF+KEEW +MGDWEK RYRNVKRNY ALIT+

AKDAFKNISIYFSKEEWTQMGDWEKIRYRNVKRNYEALITL

grayfox_Scaffold_6__1_c 7326 TAGgaggtaaatatttaggtacaggtgaactaagaaatggcaac

-0>caactaatctatcaaagcatgagaatgagatagaaacttct

cattcactcacccggagaggacggacatgtgagctaagtca

Human_NP_064612.2 65 LRATRPAFMCHRRQAIKLQ

LRA RPAFMC RR AIK Q

G:G[ggt] LRAPRPAFMCDRRWAIKPQ

grayfox_Scaffold_6__1_c 7452 GGTAACCG Intron 2 CAGGTcagcccgtatgcatgaacc

<1-----[7453 : 7817]-1> tgccgccttgagggctaca

cactactcgtccggccaca

Human_NP_064612.2 85 VDDTEDSDEEWTPRQQVKPPWMALRVEQRKHQK

VDDTEDSDEEWTPRQQ + L + K K

VDDTEDSDEEWTPRQQDEEGQEDLEISS!K-PK

grayfox_Scaffold_6__1_c 7877 gggaggtgggtacaccggggcggtgatt2a ca

taacaacaaagccgaaaaagaaatatcc a ca

atctgtttaagatggatggcgacagttt a cg

Human_NP_064612.2 118 GMPKASFSNESSLKELSRTANL

G+P+ +SN+SSLKELS TA L

GIPRVPISNKSSLKELSETAKL

grayfox_Scaffold_6__1_c 7972 GTATAGG Intron 3 AAGgacagcaaaatatagttgagat

<0-----[7972 : 10552]-0>gtcgtctgaacgtaatcaccat

aacggaatcattggagaaacgg

Human_NP_064612.2 140 LNASGSEQAQKPVSPSGEA

LN S EQ QK VS G+

LNTSSPEQGQKSVSLPGKT -:N[aat]

grayfox_Scaffold_6__1_c10619 caaaacgcgcatgtccgaaAGTACCTC Intron 4 CAGAT

tacggcaagaactctcgac <1-----[10677 : 11300]-1>

gcgtcaggggaagcttaaa

Human_NP_064612.2 159 STSGQHSRLK-LELRKKETERKMYSLRERKGHAYKEVSEPQDDDYL

T+ H ELR+K E KMYSL+ERKG AY+EVSEPQDDDYL

PTNLTHVLFSNSELRRKNVEVKMYSLQERKGLAYQEVSEPQDDDYL

grayfox_Scaffold_6__1_c11303 caacacgcttatgcaaaagggaataccgaagcgtcggagccgggtc

ccatcatttcacatggaatatatagtaagagtcaaatgacaaaaat

cccctcactccaacgagttaaggtcaaaagctacagccgcgttccc

Human_NP_064612.2 204 CEMCQNFFIDSCAAHGPPT

CE CQ FFIDSC HGPPT

Y:Y[tat] CEKCQTFFIDSCTVHGPPT

grayfox_Scaffold_6__1_c11441 TGTAAGTG Intron 5 CAGATtgatcattagatagcgcca

<1-----[11442 : 11637]-1> gaagactttaggctagccc

tggtgccctctttttgcta

Human_NP_064612.2 224 FVKDSAVDKGHPNRSALSLPPGLRIGPSGIPQAGLGVWNEASDLPLGLH

FVKDS VDKG PN SAL+LPPGLRI S IPQAGLGVWN+ASDLPLGLH

FVKDSEVDKGQPNHSALTLPPGLRIRTSSIPQAGLGVWNKASDLPLGLH

grayfox_Scaffold_6__1_c11697 tgagagggagccactgcacccgcaaaataaccggcggtaagtgcctgcc

ttaagataagacaacctctccgtgtgccgtcacgtgtgaaccatctgta

tagctaacgaactcacctgcttgacaaacctgtgtaagcgactgagtac

Human_NP_064612.2 273 FGPYEGRITEDEEAANNGYSWLI----------------

FGPYEG+ITEDEEAAN+GYSWL+

FGPYEGQITEDEEAANSGYSWLLRIIYLFPHPLASHIPP

grayfox_Scaffold_6__1_c11844 tgctggcaaggggggaagtttttaaatctccctgtcacc

tgcaagatcaaaaccaggacgttgttattcactccatcc

tcctgcacaatagacccaccgggaacccccttgcccctt

Human_NP_064612.2 296 -TKGRNCYEYVDGKDKSWANWM

TKGRNCYEYVDGKD SWANWM

ITKGRNCYEYVDGKDNSWANWM

grayfox_Scaffold_6__1_c11961 GTAGACT Intron 6 CAGaaagaattgtgggagattgata

<0-----[11961 : 12325]-0>tcaggagaaatagaaacgcagt

ccagacctgtatagtctgacgg

Human_NP_064612.2 317 YVNCARDDEEQNLVAFQYH

YVNCARDDEEQN VAFQYH

R:R[agg] YVNCARDDEEQN!VAFQYH

grayfox_Scaffold_6__1_c12392 AGGTAAGGG Intron 7 CAGGtgatgaggggca2ggtctc

<2-----[12394 : 13759]-2> atagcgaaaaaa tctaaa

tgctcgtcaggc gctatc

Human_NP_064612.2 337 RQIFYRTCRVI--RPGCELLVWYGDEYGQELGIKWGSKWKKELMAGR

RQIFYRT R CELLVWYGDEYGQELGIKWGSKWK EL AG+

RQIFYRTGRT!GHQASCELLVWYGDEYGQELGIKWGSKWKSELAAGK

grayfox_Scaffold_6__1_c13817 acattcagaa4gccgatgccgttgggtgcgcgaatgaataagcggga

gattagcggc gaacggatttgagaaagaatgtagggagagatccga

ggaccactaa ttgcccaggcgcgcgtcgagccggacgggcgcaaga

Human_NP_064612.2 382 EPKPEIHPCPSCCLAFSSQ

EP PEIHPCPSC LAFSSQ

-:A[gca] EPNPEIHPCPSCSLAFSSQ

grayfox_Scaffold_6__1_c13959 GGTGGGCA Intron 8 CAGCAgcacgacctctttcgttac

<1-----[13960 : 15746]-1> acacatacgccgctctcga

atcagatatacctgccctg

Human_NP_064612.2 401 KFLSQHVERNHSSQNFPGPSARKLLQPENPCPGDQN-QEQQYPDPHSRN

KFLSQH+E NH SQ +P S R+ +P++PCPG QN Q+QQ+ DP N

KFLSQHLEHNHPSQILPRISVREHFRPKDPCPGCQNQQQQQHSDPQRWN

grayfox_Scaffold_6__1_c15806 atcacctgcacctcacccatgagctccagctcgtcacccccctgcccta

attgaataaaaccattcgtctgaatgcaacgcggaaaaaaaacacagga

acccgtgactcttgccaaataaatcaaatacattgtggggatttagcgt

Human_NP_064612.2 449 DKTKGQEIKERSKLLNKRTWQREISRAFSSPPKGQMGSCRVGKRIMEEE

D+ KGQE KER K L K QR ISRAFS+P KGQ C I++EE

DRAKGQEGKERFKPLPKSIRQRRISRAFSTPCKGQT-TC---EGIVKEE

grayfox_Scaffold_6__1_c15953 gagagcggagatactcaaaacaaatagttactagca at ggagagg

agcagaagaagtactcagtgaggtcgctcccgagac cg agttaaa

catatagcaagcatgtataggaatagcttcgcaaaa gt gaaggag

Human_NP_064612.2 498 SRTG-QKVNPGNTGKLFVGVGISRIAKVKYGECGQGFSVKSDVITHQRT

TG QK+NP +TGKLF GVG++RI +VKY CGQGF+ +S + HQRT

PSTGSQKLNPEDTGKLFKGVGMTRIIRVKYRGCGQGFNDRSHLSRHQRT

grayfox_Scaffold_6__1_c16088 caagtcatacggagattagggaaaaaagatagtgcgtagatccaaccaa

cgcgcaatacaacgattagtgtcgttgtaaggggagtaagcatggaagc

ccacagagtagcacaacggaagaataacatactgaccttgatccatgga

Human_NP_064612.2 546 HTGEKLYVCRECGRGFSWKSHLLIHQRIHTGEKPYVCRECGRGFSWQSV

HTGE YVCRECGRGF+ +++L+IHQR HTGEKPYVCREC GF+ +

HTGENPYVCRECGRGFTHRTNLIIHQRTHTGEKPYVCRECRXGFTERLT

grayfox_Scaffold_6__1_c16235 caggactgtagtgcgtacaaacaaccaacaggactgtagtatgtagata

acgaacatggaggggtcagcatttaagcacgaacatggaggggtcagtc

cgagctttcggtgactacaattcatgaacagggcttcgatgactaggat

Human_NP_064612.2 595 LLTHQRTHTGEKPYVCRECGRGFSRQSVLLTHQRRHTGEKPYVCRECGR

L HQRTHTGEKPYVCR+CGRGF+++S L HQR HTGEKPYVCRECGR

LNEHQRTHTGEKPYVCRKCGRGFTQRS!LSEHQRTHTGEKPYVCRECGR

grayfox_Scaffold_6__1_c16382 cagccaacaggactgtaatgcgtacat1cagccaacaggactgtagtgc

taaaagcacgaacatggaggggtcagc tgaaagcacgaacatggaggg

ccacggacagggcttcaatgactagga ccacggacggggcttcggtga

Human_NP_064612.2 644 GFSRQSVLLTHQRRH

F+++S L THQR H

SFTKRSTLSTHQRTH

grayfox_Scaffold_6__1_c16527 ataaatacaaccaac

gtcagcctgcaagca

ctaggatccacggac

**Figure S1. *PRDM9* alignment output from Genewise**. Alignment using human protein sequence and Urocyon DNA sequence. The four frameshift mutations (indicated by !) a premature stop codon (indicated by X) are highlighted in red. The homology highlighted in yellow was seen across canid species analyzed in previous work (Mooney et al. 2023 & Axelsson et al. 2012).
